# Galston’s Resume of Eugenics

**Published:** 1915-03

**Authors:** 


### Galston's Resume of Eugenics.

"Individuals appear to me as partial detachments from the infinite ocean of being and this world as the stage on which evolution takes place, principally hitherto by means of natural selection, which achieves the good of the whole with scant regard to that of the individual.

"Man is gifted with pity and other kindly feelings; he also has the powers of preventing many kinds of suffering. I conceive it to fall well within his province to replace natural selection by other processes that are more merciful and not less effective.

"This is precisely the aim of eugenics. Its first object is to check the birth rate of the unfit, instead of allowing them to come into being, though doomed in large numbers to perish prematurely. The second object is the improvement of the race by furthering the productivity of the fit by early marriages, and healthful rearing of their children. Natural selection rests upon excessive production and wholesale destruction; eugenics on bringing no more individuals into the world than can be properly cared for, and those only of the best stock."

